# Child Torture in Christian England

**Published:** 1887-01-15

**Authors:** 


### CHILD-TORTURE IN CHRISTIAN ENGLAND.

UNDER the Roman law, fathers had power of life and death over their children. It was within their right to inflict torture upon them, to sell them as slaves, or to cast them out to die. Having neither fear of God, nor love for man, they were ignorant whether they or their children had souls to be saved or lost. A Roman recognised no obligation towards his children, and held them to have no rights of their own. Christ brought a different view of life and its responsibilities into a world of darkness. He taught the doctrine of love. A love of fatherhood revealed in the Eternal Father, as well as the brotherhood of man; and the sanctity of home. In nothing was the sympathy of Christ shown more touchingly than in the love which He displayed towards children; He took them up in His arms, He laid His hands upon them, and blessed them.

The contrast between the Roman and Christian dispensations and their treatment of children should be borne in mind in considering the case of Dr. Williams and his wife, whose ill-treatment of their little child, Gladys, has excited so much commiseration and sympathy during the last week. There is evidence that such acts of criminal cruelty to children are not confined to the lowest and most degraded classes of the people, but are chiefly to be met with amongst those in receipt of good wages, or possessing independent means. It is sad to reflect that out of 147 cases of cruelty of the worst kind during the past year, in one-third of the instances the mothers were the assailants. Step-fathers and mothers show less cruelty to children than public officials, despite the popular belief to the contrary; and we regret to state that there were seven charges of cruelty against nurses brought before the magistrates. The ages of the children varied from one month to over twelve years, and it seems that the younger the child the more brutal and horrid becomes the assault of the cowardly ruffians of both sexes who disgrace their race by the hateful deeds of inhumanity of which they are the perpetrators.

The report of the London Society for the Prevention of Cruelty to Children, cannot be read by any humane parent without pain and indignation of the most aggravated kind. It contains an account of the sufferings of 327 small children, of whom forty were savagely assaulted with boots, fists, thongs of rope, pokers, knives, pieces of timber; with anything, indeed, which came readiest to the furious hand. The parents of twenty-seven children determined to be rid of their offspring, and with this view commenced a system of gradual starvation and deliberate exposure with a designed and direct intention to kill. Many children were subject to culpable and serious neglect, being left for the whole day locked up and without food; and, in some cases, bound and gagged, and confined in small boxes, without even water to drink or the power to get at it, had it been there. We hope every one of our readers will obtain this report, and study it carefully.

Our mission is to awaken an interest in the institutions which provide for the sick and suffering, and in this connection it will be our duty from time to time to examine into the causes which lead to much of this illness. We have been writing of direct cruelty to children in out-of-the-way places, or, at any rate, in places hidden from the public gaze. Unfortunately, these are not the only cases of child-torture in England, nor indeed are they the worst. The thoughtless, but kind-hearted and well-to-do wayfarer in the public streets, has his sympathies awakened by the piteous supplications of beggars, accompanied by gaunt, starving and emaciated children. The child torturer knows full well the effect of such an exhibition, and he proceeds to fill his purse, by compelling weary, pale, and cold little frames, many of them dying from want, neglect and cruelty, to stand for hours in the public thoroughfares in rain, or at night, until his greed has been satisfied. Seventeen such cases, several of which have caused the death of the children, have been dealt with by the Society during the year. A correspondent sends particulars of a girl and boy, aged eight and five years respectively, who supported six adults in idleness for months, until through fear of arrest it was stopped. Amidst the snows of winter these little ones could be seen with feet exposed, with earnest appeal (which meant a whipping if they were not successful) going their daily round, and bringing in their spoils to the lazy men and women awaiting their return, who would gorge themselves with what they chose from the booty secured. Who were guilty of this cruelty to children—the parents? Undoubtedly. The neighbours who shared in this heartless business? Most assuredly. Who else? Who but those who gave the broken food and money. Not consciously so, but really accomplices in the sin. We wonder if it ever occurred to those who give to such cases—many of them being, as we know, tender-hearted, charitably-disposed people—that the very fact of the children coming to their houses or begging in the streets, is proof that there is a fearful wrong somewhere, and that the only true charity is to search out that wrong, and, if possible, correct it.

It is true, of course, that further legislation is greatly needed in the direction of empowering the authorities in the Metropolis to deal with these cases in the same way that they are treated in Glasgow, Birmingham, Liverpool, and elsewhere. The real remedy—the best—will, however, be found in an awakening of the public conscience and of the individual will, for then it will not be possible for a girl and a boy to support a family of six in utter idleness by begging. Would that each individual would devote sufficient time to inquire into a case before giving to it. Those who have observed what is going on around them, must be aware that in certain leading thoroughfares like Regent Street, Westbourne Grove, and Oxford Street, the same groups of child-torturers and the tortured may be seen soliciting alms from time to time throughout the year. We hear of a little Jewish girl in Westbourne Terrace who has been made to beg in this way in all weathers for months, and who may be seen there by anyone to-day. She seems to be under the control of a wretch who keeps the child at work for many hours, with the view apparently of providing her with money to spend in drink and dissipation. There are no doubt hundreds of similar cases in London; but if the casual passer-by will, for the future, search out the wrong, instead of salving his conscience by feeding the torturers at the expense of the tortured, such cases will speedily disappear from our streets.

Our readers are drawn from all parts of the country as well as from the Metropolis, and we are hopeful, therefore, that by calling attention to the child-torture which exists in Christian England to-day, we may awaken many to a determination to spend and be spent in its suppression. The Master said, remember, "Suffer little children to come unto Me, and forbid them not, for of such is the Kingdom of Heaven."

We shall be glad to receive offers of help or of money for the London Society for the Prevention of Cruelty to Children, 7, Harpur Street, Theobald's Road, London, W.C. The Secretary, Dr. Buxton, will send a report on application.

